# Surgical Treatment of Diphtheria

**Published:** 1890-05-17

**Authors:** 


### SURGERY.

### SURGICAL TREATMENT OF DIPHTHERIA.

The question whether or not tracheotomy should be performed for diphtheria, once much debated, appears now to be decided in the affirmative. But the question, at what period of the progress of the malady it should be undertaken, is not quite so positively fixed. It is laid down in a standard work that "if this one chance remain in any degree, however small, he" (the medical attendant) "is bound to offer it to his patient." The simple question is, does tracheotomy give the smallest chance of life to the patient who without tracheotomy must inevitably die from asphyxia? "The answer to this question may, as a rule, be given in the affirmative, if the obstruction be not below the situation in which tracheotomy is performed." This, however, cannot, especially with children, be always clearly ascertained. When affection of the bronchial tubes or pneumonia exists, or if the patient is phthisical, the probability of saving life by tracheotomy is smal'. Practitione:s are not, however, wanting who advocate early surgical treatment in diphtheria. And it is quite certain that a cure is more likely to follow the operation if performed before the system becomes impregnated, and exhaustion prominent. For there is in diphtheria not only the peril of death from suffocation, but also danger from prostrating toxæmia and paralysis of the heart. It is asserted that these latter sequences may be prevented by the admission of a sufficient quantity of air, while the tendency to death by suffocation may be removed. We have now corroborative evidence from the Antipodes, where diphtheria is quite as common, if not more so, than in this country. Dr. C. P. Clubbe, surgeon to the Sick Children's Hospital, Sydney, publishes remarks on the treatment of diphtheria by tracheotomy. The author agrees with Mr. Watson Cheyne as to the desirability of early operation. He operates first to prevent, or to try to prevent the spread of the membrane down the trachea; and secondly, to prevent the child being choked, and not, as the writer in "Holmes' System of Surgery" observes, to give relief from impending suffocation. Mr. Clubbe believes in using the knife right down to the trachea. He disregards the isthmus of the thyroid, but he nearly always cuts below, as he desires to open the trachea as low down as possible. When the trachea is once open, blunt hooks should be introduced and the cut edges held apart, so that a thorough inspection may be made. The tubes he uses are the ordinary bivalve ones. But, as much depends on the after treatment as on the operation. After eight or twelve hours an inspection should be made under chloroform, and any membrane should be removed, or if not easily removable, dissolved by a solution of trypsin 15 grains, with 20 grains of sodæ bicarb., in 2 oz. of water. After the trachea has been cleared it should be touched with a solution of perchloride of mercury, 1 in 500. The author next gives a very good piece of advice to those who follow his treatment. This is to be provided with a second set of tubes, for the two sides of the tracheal wound may come together, curve inwards, and a catastrophe may ensue. The tubes should be taken out about every eight hours so long as membrane is found in the trachea. When attending to such cases it is best to wear a white linen coat, and, if possible, to protect the face in some way, for children always cough out mucus in all directions. A laryngeal mirror on the forehead facilitates matters at night. It is almost useless to do a tracheotomy for diphtheria unless there are two good nurses to watch the case afterwards, for children sometimes tear the tube out, and sudden symptoms of dyspnœa may arise from various causes. The tubes should be left off altogether as soon as membrane has ceased forming, and the child can breathe with the opening stopped up. The average time is about twelve days. Some authors, while giving minute directions for the performance of tracheotomy and the dressing of the wound, do not advise change of the tube. "Formerly," Mr. Clubbe observes, "the surgeon used to consider his work was at an end when he had put the tube in. The tube was put in and left there till the child died. If the membrane did not go down the trachea all the better, and probably the child recovered. But if the membrane got down the surgeon made little or no attempt to get it up or prevent its spreading, but shrugged his shoulders and walked off." We are not aware that Mr. Clubbe's plan is advocated in any of the writings on diphtheria in this country, but although there may be objections to frequently interfering with the wound in the way described, there certainly may be great benefit from thus freeing the parts from accumulation or fresh formation of membrane. With reference to the desirability of early operation in diphtheria, we think that if our present method of treatment is not correct there is another reason in favour of surgical interference. We refer to a recent writer on this subject. Mr. F. W. Elsner, of Melbourne, has some remarks in the last number of the *Australian Medical Gazette* to hand on the irrationality of the treatment of diphtheria by steam. Mr. Elsner believes that the micrococcus is the vehicle of infection, and that it must be present to constitute true diphtheria. But when we proceed to make cultures one of the first things required is a suitable temperature and moisture. Consequently if we by playing warm moist vapour upon a hotbed of micrococci, such as is presented by a diphtheritic membrane, we furnish the essentials necessary for their multiplication. The addition of an antiseptic does not neutralize the evil consequences of such unscientific treatment, for were the antiseptic sufficiently strong to kill the micrococci it would likewise kill the patient by absorption. The author advises the use of an antiseptic in glycerine, for glycerine abstracts moisture from the membrane and prevents the antiseptic from sinking into the mucous membrane and so poisoning the individual; and the best antiseptic is, he thinks, carbolic acid. There is also another objection to the use of steam. It washes into the larynx soluble and infective constituents of the diphtheritic membrane, notably the micrococci, which are caught at the vocal cords and forced to breed from being fed continuously from above. In another part of his paper Mr. Elsner observes that the micrococci of diphtheria after a time invade the circulation, develop ptomaines, and the usual phenomena of septicæmia, or pyæmia. But some time ago Mr. Watson Cheyne, in a paper on Tracheotomy in Diphtheria, published in the *British Medical Journal*, quoted Loeffler's researches, which tend to show that the bacilli do not penetrate into the circulation, so that the disease from first to last remains a local one, and the general symptoms are due to chemical poisoning. However this may be, Mr. Elsner's views should demand attention and consideration, for hot, moist airs are advised not only after the operation of tracheotomy, but also as a remedial measure in diphtheria. Some time back in The Hospital (December 14th, 1889) we noticed the treatment of diphtheria as advocated by Dr. Murray Gibbs and others in Australia by the vapour of the eucalyptus leaves conveyed by steam. Marvellous results were reported, and the curative properties of the eucalyptus were thought to be such as would eventually do away with any necessity for surgical procedure. We then observed that the throat affection one physician would dignify by the name of diphtheria, another physician might designate by a more homely title. Diphtheria differs much in intensity, from the mildest form, which resembles an ordinary catarrhal sore throat, to the most severe character, when the malady assumes a gangrenous, or putrid, form. This variability of the affection leads to very different reported results in the practice of physicians. A reported cure of sixty-two cases led to the suspicion that the author was liberal in the use of the term diphtheria. Respecting the above, we now observe that Mr. H. W. Elsner, of Melbourne, says in the last number of the *Australian Medical Gazette* to hand, that the discussion on this treatment which ensued elicited nothing further than a general dubiousness as to the accuracy of the diagnosis, the general impression being that the four hundred cases said to have been treated in a short period at Taranaki and elsewhere could not have been true cases of diphtheria. With this conclusion Mr. Elsner fully agrees. While on this subject, we may mention that it should not be forgotten that diphtheria may develop itself on any mucous membrane. Dr. Millikan has a paper in the *Philadelphia Medical and Surgical Reporter* for February last on diphtheria of other parts, and Jacobi gave an account of a case in which the bladder was found lined with diphtheritic membrane. But many instances have also been noticed of the appearance of the disease on the common integument when it has been denuded by any cause, as vesication for instance, of its external layer. It is not unknown behind the ears of children, and it takes root freely at the angles of the mouth, and at the palpebral angles, where the skin merging into mucous membrane, is liable to ulceration or abrasion. In these latter conditions Dr. Spohn's treatment by turpentine spray is worthy of trial. (Vide Hospital Retrospect, December 14th, 1889.)

